# Supplementary material for: Automated identification and tracking of cells in Cytometry of Reaction Rate Constant (CRRC)
Source: PLoS One. 2023 Jul 3;18(7):e0282990. doi: 10.1371/journal.pone.0282990 (PMC10317225; doi:10.1371/journal.pone.0282990)
Supplement: S1 File — (PDF) [file pone.0282990.s001.pdf]

SUPPORTING INFORMATION

Automated identification and tracking of cells in cytometry of reaction rate constant (CRRC)

Giammarco Nebbioso[1,2¶], Robel Yosief[1,2¶], Vasilij Koshkin[1,2], Yumin Qiu[2,3], Chun Peng[2,3], Vadim Elisseev[4,5], and Sergey N. Krylov[1,2*]

[1] Department of Chemistry, York University, Toronto, Ontario, Canada

[2] Centre for Research on Biomolecular Interactions, York University, Toronto, Ontario, Canada

[3] Department of Biology, York University, Toronto, Ontario, Canada

[4] IBM Research Europe, The Hartree Centre, Daresbury Laboratory, Warrington, United Kingdom

[5] Wrexham Glyndwr University, Wrexham, United Kingdom

*** Corresponding author**

E-mail: skrylov@yorku.ca (SNK)

¶ These authors contributed equally

TABLE OF CONTENTS

Supplementary Files Name and Description **S2**

Note S1: Microscope Settings and Microscopy Protocol **S4**

Note S2: Fiji and TrackMate Detailed Workflow **S5**

Note S3: Robustness of CRRC to Cell Diameter **S7**

Figure S1. Illustration of robustness of CRRC to a range of cell diameters. Kinetic histograms of k_{efflux} rate constants found in TOV-112D cells using 10, 15, and 20 μm cell diameters are shown S7

Note S4: Distribution of Cell Migration Speeds **S8**

Figure S2. Distribution of cell migration speeds (μm/h) found by high frequency (1 image per 10 s) time-lapse BF imaging S8

Note S5: Determination of Error in Fluorescence Intensity Integration Caused by Shift in Cell Position from the Fluorescence Image and Accompanying BF Image **S9**

Figure S3. Schematic representation of the effects of cellular movements on fluorescence integration. S9

Note S6: Consideration of Focusing for Fluorescence Intensity Integration S10

Figure S4. Relative deviations in intracellular fluorescence intensities at different Z positions ... S10

Note S7: Comparison of fluorescence-decay kinetic curves in the original and new workflow S11

Note S8: Considerations on statistics S13

Additional supplementary files can be found on (see version 1 for both data and text):
10.6084/m9.figshare.20152820

File name and description

- images.zip
  - It contains raw images for:
    - Figure 2 (main text); the main folder contains two subfolders, 'Plastic-bottom dish' and 'Glass-bottom dish'. Within the 'Plastic-bottom dish' subfolder, there are three more subfolders named 'region X'. Within the 'Glass-bottom dish' subfolder, there are five more subfolders named 'region X'. Each region represents a different *x-y* position of the cell plate. Each 'region X' folder contains images that were obtained in fluorescence, BF, DIC and PC modes, respectively.
    - Figure 3 (main text); it contains raw images for the time lapse experiment. The time interval between each image is 10 s.
    - Figure 6 (main text); it contains the set of adjacent BF and fluorescent images obtained during the CRRC experiment. The time interval between the images in the BF folder is 1 min. The time interval between the images in the Fluo folder is 1 min. The folder also contains an image named 'PI'. This image reveals the position of each single cell at the end of the CRRC experiment upon PI staining, and it is used in the original workflow.
    - Figure S1 (SI); the main folder contains three subfolders named 'region X'. Within 'region X' folders are a set of adjacent BF and fluorescent images obtained during the CRRC experiment. Region 1 is the same images used in Figure 6.
    - Figure S4 (SI); this folder contains two BF images: the raw image and the image after threshold was applied. It also contains the in-focus and out-of-focus (5 µm below and 5 µm above the in-focus position) fluorescence images.
- Kinetictraces_and_fittingresults.zip
  - Figure 6 (main text) and Figure S1 (SI); It contains five .csv files. The .csv files are named 'Kinetic traces_and_fittingresults_new workflow', 'Kinetic traces_and_fittingresults_original workflow', 'Kinetic traces_and_fittingresults_10 µm cell diameter', 'Kinetic traces_and_fittingresults_15 µm cell diameter', and 'Kinetic traces_and_fittingresults_20 µm cell diameter'.
    All five files contain one sheet displaying the fluorescence intensity values as a function of time for individual cells. It also contains the equation used for fitting and the fitting results computed by OriginPro (scroll down to row 69).
- trackingvideos.zip
  - Figure 5 (main text) and Note S7 (SI); It contains three .mp4 files. For the highly motile cell in fig.5A, the videos show the difference between the cell mask and the true cell position during the time-lapse experiment. The three videos are named: 'original' (showing the inability of the workflow to track the cell), 'new' (showing the tracking ability of the new workflow for the same cell) and 'combined' (this is a comparison between the cell masks of both workflows).

- kineticcurve_comparison.zip
    - Note S7 (SI); It contains five .pptx files. In all five files, each slide displays the original and new workflow results, including kinetic constant, standard error, fitting status, and kinetic curve for the kinetic curve of a single cell. The file named ‘Total_67_Cells’ displays the results for all 67 cells analyzed with both workflows. The file titled ‘Filtered_47_Cells’ includes the results for cells that successfully fitted the exponential decay function in both workflows. The file named ‘Original_Workflow_Overestimate’ includes cells from the remaining 47 cells, in which original workflow overestimated the kinetic constant. The file labeled ‘New_Workflow_Overestimate’ includes cells from the remaining 47 cells, in which new workflow overestimated the kinetic constant. The file titled ‘Equal_Kinetic_Constant’ includes cells from remaining 47 cells that had the same kinetic constant under the new and original workflow.

Note S1: Microscope Settings and Protocol
Imaging was performed with a Leica DMi8 high-throughput cell-imaging system. Four modes were used: fluorescence, brightfield (BF), differential interference contrast (DIC), and phase contrast (PC). The 'Mark and Find' feature of the microscope was used to acquire images of multiple regions of the cell plate and the 'Relative Focus Correction' feature was used to set different Z-positions between the fluorescence and BF channels. Image settings for each figure are shown below:

Figure 2 in the main text
- BF: no binning, 7.81 ms exposure, high well capacity, intensity 48, aperture 7, transmitted light field diaphragm (Tl-Fld) 23, 196–191 intensity threshold
- DIC: no binning, 7.81 ms exposure, high well capacity, intensity 128, aperture 15, Tl-Fld 46, bias 50, 192–192 intensity threshold
- PC: no binning, 7.81 ms exposure, high well capacity, intensity 130, aperture 24, Tl-Fld 23, 129–128 intensity threshold
- Fluorescence: RHOD channel, no binning, 50 ms exposure, low noise, fluorescence intensity manager (FIM) 30%, incident light field diaphragm (Il-Fld) 6

Figure 3 in the main text
- BF: no binning, 8 ms exposure, high well capacity, intensity 48, aperture 11, Tl-Fld 46, 84–80 intensity threshold

Figures 5, 6 in the main text and Figure S1 in the supporting information
- BF: no binning, 7.81 ms exposure, high well capacity, intensity 38, aperture 12, Tl-Fld 46, 196–191 intensity threshold
- Fluorescence: FITC channel, no binning, 7.81 ms exposure, low noise, FIM: 30%, Il-Fld 6

Figure S4 in the supporting information
- BF: no binning, 7.81 ms exposure, high well capacity, intensity 48, aperture 7, Tl-Fld 23, 196-191 intensity threshold
- Fluorescence: Y5 channel, 2×2 binning, 50 ms exposure, low noise, FIM 30%, Il-Fld 6

Note S2: Fiji and TrackMate Detailed Workflow

Here we explain the new workflow step-by-step. The time-lapse fluorescence and BF images, which are archived in images.zip, were evaluated in nine steps, as shown below. Image processing was conducted using the Fiji software (https://imagej.net/software/fiji/). Cells were tracked with TrackMate (version 7.6.1; https://imagej.net/plugins/trackmate/), an open-source plugin available on Fiji.

1. Load both sets of images (BF and Fluo) onto Fiji

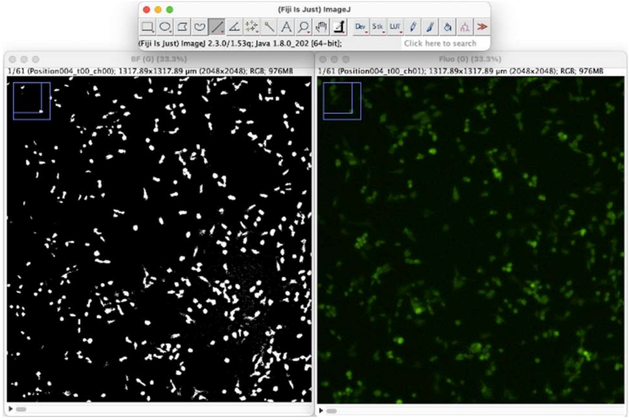

2. Merge the BF and Fluo images as a composite. Assign BF as Channel 1 and Fluo as Channel 2

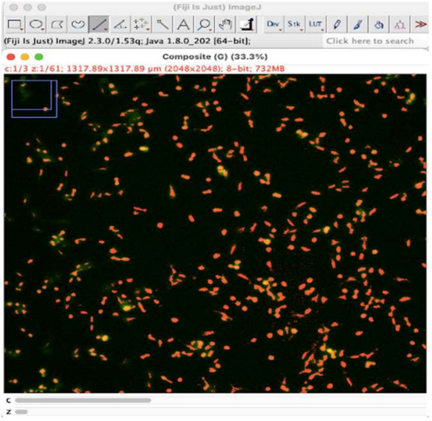

3. Use the Fiji plugin TrackMate on the composite channel

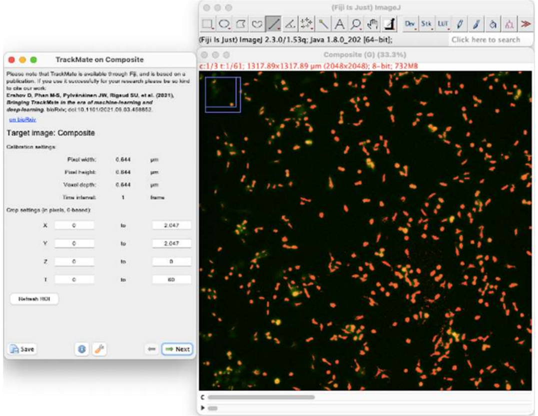

4. Select StarDist detector for cell segmentation on channel 1 (BF)

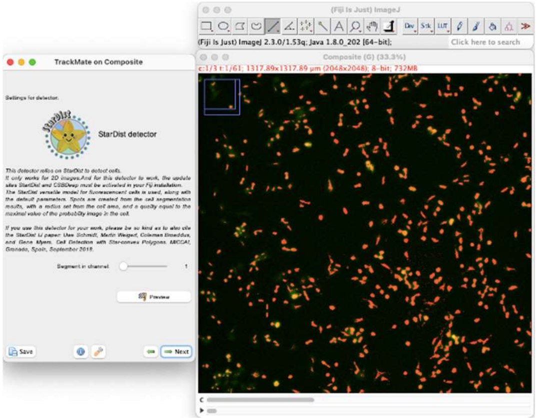

5. Apply radius range filter

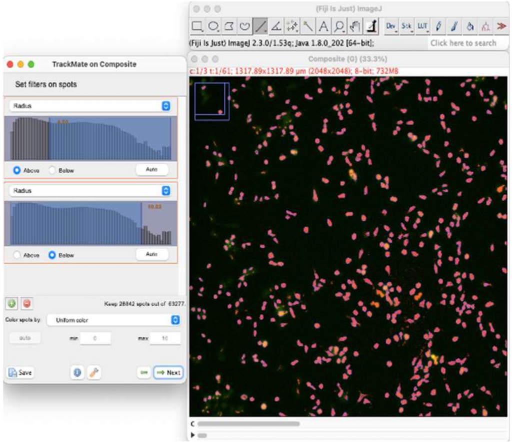

6. Select LAP tracker for cell tracking

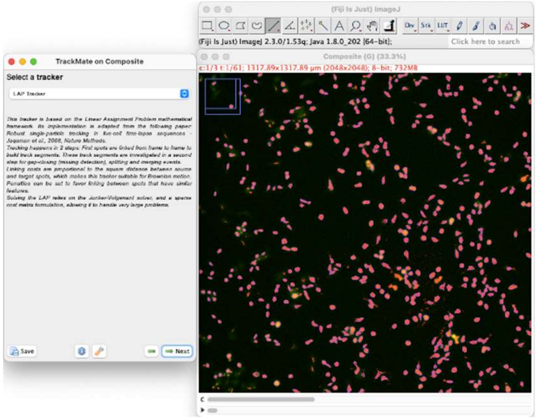

7. Enter max distance that cells can travel between frames (~ cell diameter) and do NOT allow gap closing, merging, or splitting

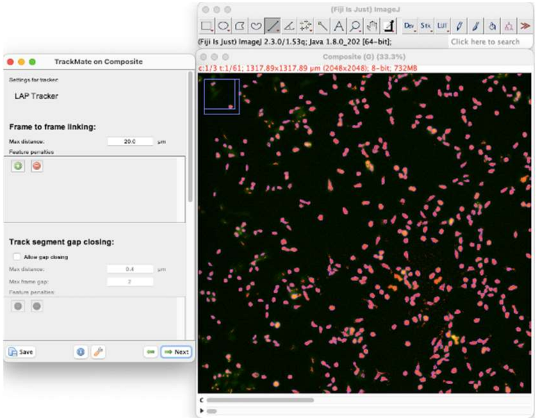

8. Filter the generated cell tracks by the duration of track, include only full-length tracks

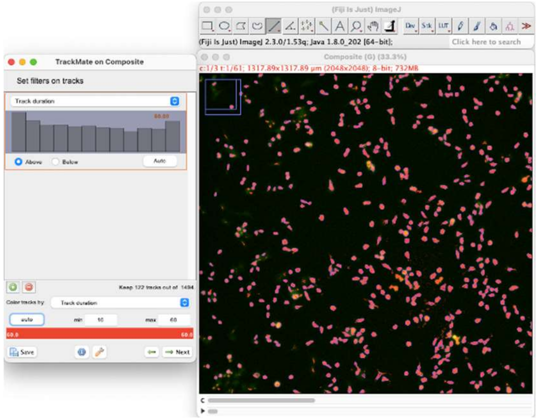

9. Open "Track tables" and select spots tab. Adjust Track IDs (cells) column so that cells are sequentially ordered by frame # and click export to CSV. In the CSV file, only the "Track ID" and "Mean Intensity Channel 2" columns are kept (highlighted red).

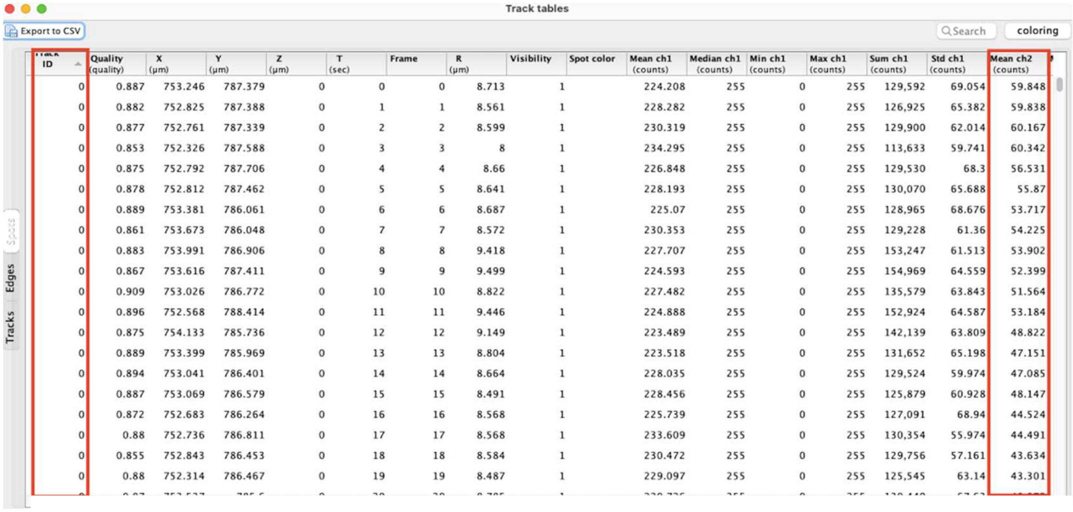

Track ID	Quality (quality)	X (µm)	Y (µm)	Z (µm)	T (sec)	Frame	R (µm)	Visibility	Spot color	Mean ch1 (counts)	Median ch1 (counts)	Min ch1 (counts)	Max ch1 (counts)	Sum ch1 (counts)	Std ch1 (counts)	Mean ch2 (counts)
0	0.887	753.246	787.379	0	0	0	8.713	1		224.208	255	0	255	129,592	69.054	59.848
0	0.882	752.825	787.388	0	1	1	8.561	1		228.282	255	0	255	126,925	65.382	59.838
0	0.877	752.761	787.339	0	2	2	8.599	1		230.319	255	0	255	129,900	62.014	60.167
0	0.853	752.326	787.588	0	3	3	8	1		234.295	255	0	255	113,633	59.741	60.342
0	0.875	752.792	787.706	0	4	4	8.66	1		226.848	255	0	255	129,530	68.3	56.531
0	0.878	752.812	787.462	0	5	5	8.641	1		228.193	255	0	255	130,070	65.688	55.87
0	0.889	753.381	786.061	0	6	6	8.687	1		225.07	255	0	255	128,965	68.676	53.717
0	0.861	753.673	786.048	0	7	7	8.572	1		230.353	255	0	255	129,228	61.36	54.225
0	0.883	753.991	786.906	0	8	8	9.418	1		227.707	255	0	255	153,247	61.513	53.902
0	0.867	753.616	787.411	0	9	9	9.499	1		224.593	255	0	255	154,969	64.559	52.399
0	0.909	753.026	786.772	0	10	10	8.822	1		227.482	255	0	255	135,579	63.843	51.564
0	0.896	752.568	788.414	0	11	11	9.446	1		224.888	255	0	255	152,924	64.587	53.184
0	0.875	754.133	785.736	0	12	12	9.149	1		223.489	255	0	255	142,139	63.809	48.822
0	0.889	753.399	785.969	0	13	13	8.804	1		223.518	255	0	255	131,652	65.198	47.151
0	0.894	753.041	786.401	0	14	14	8.664	1		228.035	255	0	255	129,524	59.974	47.085
0	0.887	753.069	786.579	0	15	15	8.491	1		228.456	255	0	255	125,879	60.928	48.147
0	0.872	752.683	786.264	0	16	16	8.568	1		225.739	255	0	255	127,091	68.94	44.524
0	0.88	752.736	786.811	0	17	17	8.568	1		233.609	255	0	255	130,354	55.974	44.491
0	0.855	752.843	786.453	0	18	18	8.584	1		230.472	255	0	255	129,756	57.161	43.634
0	0.88	752.314	786.467	0	19	19	8.487	1		229.097	255	0	255	125,545	63.14	43.301

The CSV file is then opened in Microsoft Excel, the time dependencies of fluorescence intensities (kinetic traces) for each cell are arranged to be side-by-side for exponential fitting. The CSV files containing the traces can be found in kinetictraces_and_fittingresults.zip.

Note S3: Robustness of CRRC to Cell Diameter

From the CRRC cross-membrane experiment (Figures 5 and 6 in the main text), cells were re-analyzed using the Laplacian of Gaussian (LoG) detector within TrackMate. With this detector, cells are recognized based on an estimated diameter that can be varied. The estimated cell diameters were 10, 15, and 20 µm. It was found that the k_{efflux} distributions were not significantly different, according to the Kolmogorov-Smirnov test at the 0.05 level.

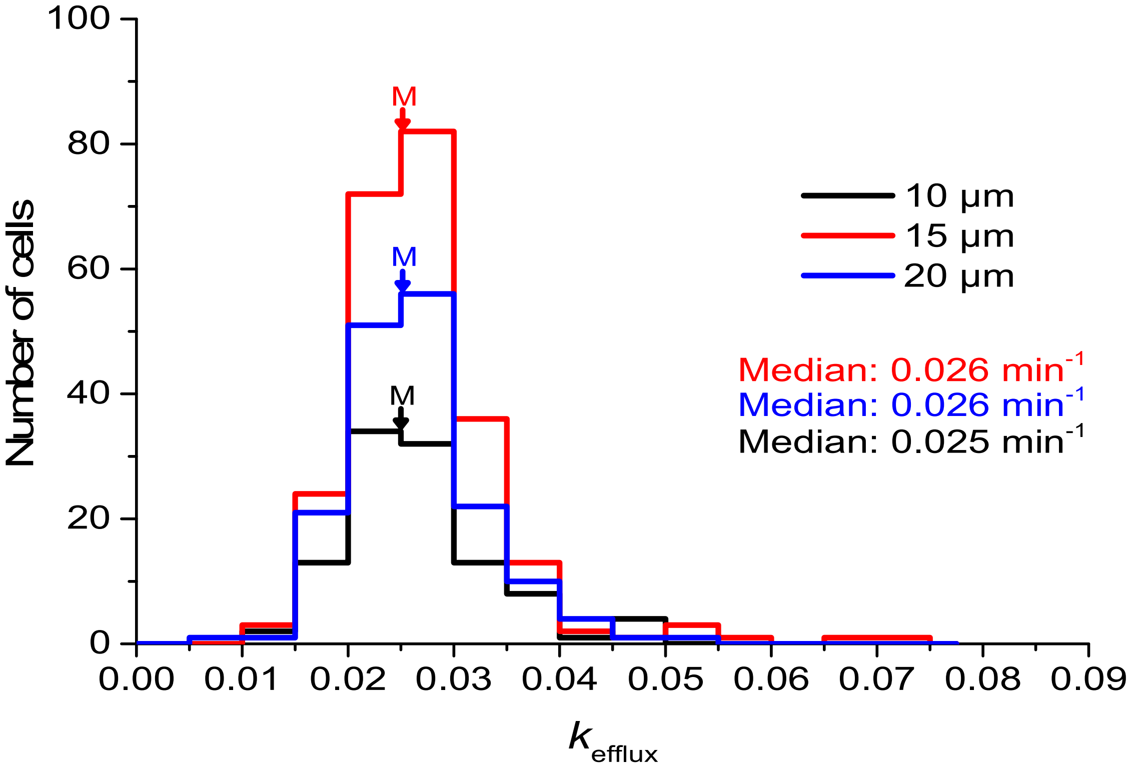

Figure S1. Illustration of robustness of CRRC to a range of cell diameters. Kinetic histograms of k_{efflux} rate constants found in TOV-112D cells using 10, 15, and 20 µm cell diameters are shown. The variation in sample size occurred to differences in cell identification and filtering; however, each distribution consisted of over 100 cells. Median k_{efflux} values are displayed and their positions are indicated by the arrows. The k_{efflux} distributions were compared using the Kolmogorov-Smirnov test and were found not to be significantly different at the 0.05 level. The p values were 0.66 (10 and 15 µm), 0.82 (10 and 20 µm), and 0.81 (15 and 20 µm).

Note S4: Distribution of Cell Migration Speeds

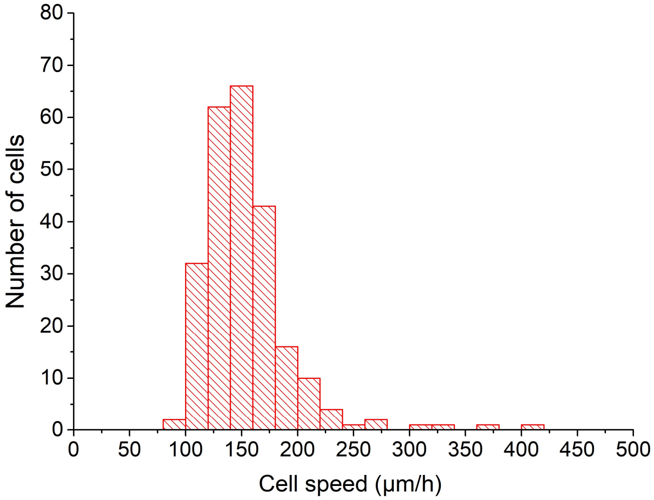

Figure S2. Distribution of cell migration speeds (μm/h) found by high frequency (1 image per 10 s) time-lapse BF imaging. The distribution was not normal according to the Shapiro-Wilks normality test at the 0.05 level ($P = 2.2 \times 10^{-16}$). The peak of the distribution was approximately 150 μm/h with an interquartile range of 40 μm/h. The fastest cell had a speed of approximately 400 μm/h.

Note S5: Determination of Error in Fluorescence Intensity Integration Caused by a Shift in Cell Position from the Fluorescence Image and Accompanying BF Image

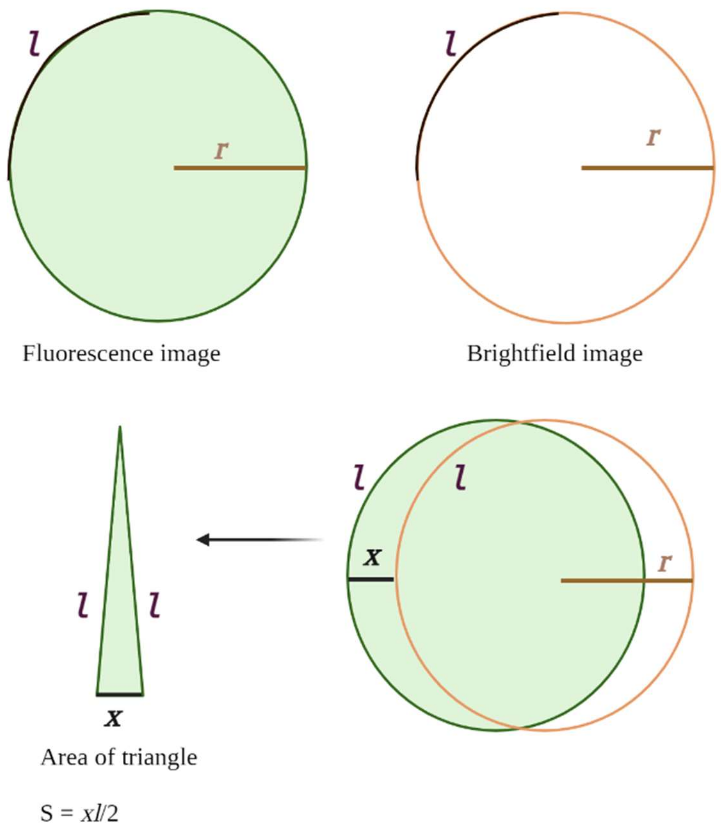

Figure S3. Schematic representation of the effects of cellular movements on fluorescence integration. Let's assume that $x/r << 1$, where x is the distance travelled by the cell and r is a cell radius. In this case, the area of the lune encompassing x can be approximated by that of a triangle: $s = xl/2$. The total area that is excluded from the overlap of the two circles is $S = 2s = 2xl/2 = xl = x(2\pi r/4) = \pi xr/2$. The area of the circle is: $S_{circle} = \pi r^2$. The area of the shape of overlap of the two circles is the one that will be used for fluorescence intensity determination. It is smaller than the area of a single circle by S. The relative error of circle area determination is: $\Delta S = S/S_{circle} = \pi xr/(2\pi r^2) = x/(2r) = x/d.$

Note S6: Consideration of Focusing for Fluorescence Intensity Integration

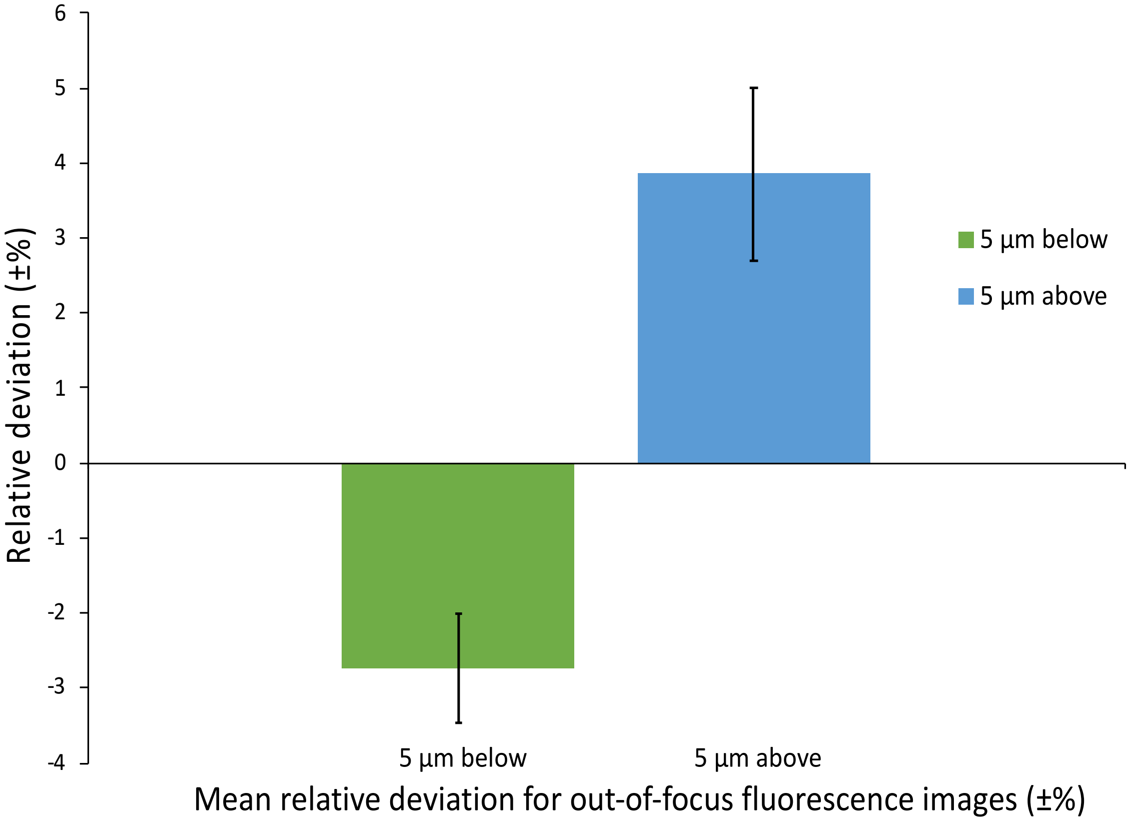

Figure S4. Mean relative deviations with 95% confidence intervals (CI) in fluorescence intensities at different Z positions. Cells were stained with a 2 µM cytoplasmic probe DRAQ9 solution (Novus Biologicals, Littleton, CO, USA, Cat. No: NBP2-81128) in HBSS. After 30 min, cells were washed three times with PBS (Cytiva, Logan, Utah, USA, Cat. No: SH30256.01). Cell contours were determined from taking a high-contrast BF image. An in-focus fluorescence image (Cy5 cube) was taken for reference and fluorescence images 5 µm below and 5 µm above the reference were taken . Intracellular fluorescence intensities obtained from the in-focus image were used to find relative deviations in intensities for the out-of-focus images. The mean relative deviations were -2.2% (95% CI, -1.46% – -2.94%) for the fluorescence image taken 5 µm below and 3.6% (95% CI, 2.4% – 4.8%) for the fluorescence image taken 5 µm above the reference.

Note S7: Comparison of fluorescence-decay kinetic curves in the original and new workflow
This note aims to analyze in detail the kinetic curves shown in Figure 5 of the main text. Figure 5 shows the kinetic curves corresponding to cells with low and high motility. Such kinetic curves were obtained with both the original and new workflow. For the high-motility cell, the kinetic curve determined from the new workflow was an expected exponential decay, since the cell was followed during the time-lapse experiment (a .mp4 file showing this event titled 'new' can be found in trackingvideos.zip). However, for the same highly-motile cell (Figure 5A), the original workflow recorded an unusual initial increase in fluorescence intensity, followed by a quick decay. This unusual kinetic behaviour should be ascribed by the cell-contour mask used for fluorescence integration. In the original workflow, the contour mask is obtained at the end of the time lapse experiment. It is noteworthy that such a mask often differs from the true cell position at the end of the time-lapse experiment. This is due to the addition of PI (necessary to obtain the mask), which occurs after the time-lapse experiment is completed and often causes an additional last shift in cells' positions. As a result, the true cell location in the initial stages of the experiment will be mainly outside the boundaries of the cell-contour mask; hence, little to no fluorescence will be integrated. However, as time goes by, the cell will move and its true location will start overlapping with the position suggested by the cell-mask; as a result, a larger fluorescent area will be integrated, explaining the initial fluorescence increase. In our case, at approximately 40 min, the cell stopped migrating; consequently, the overlapping process between the true cell position and the cell-mask ceased and the increase in fluorescence intensity stopped. Then, it becomes clear that the sudden drop in fluorescence observed after 40 min is the only portion of the kinetic data that can be attributed to cross-membrane activity (a .mp4 file demonstrating this phenomenon titled 'original' can be found in trackingvideos.zip). As explained in the main text, the main observation of our comparative study of cells with different motility is that the original workflow tends to overestimate the rate constant of substrate efflux for high-motility cells. To confirm our remark, we decided to compare the kinetics of all single cells that were analyzed by both the original and new workflow. A total of 67 cells were used for this comparison, both with high and low motility. The major results from this comparative study are:

1. For the original workflow, 20 out of 67 cellular kinetics failed the exponential decay fitting. For the new workflow, only 2 cellular kinetics failed the fitting step. Such cells were filtered out hence they did not influence the histogram (Figure 6 main text).
2. In total, the cellular kinetics of 47 out of 67 cells successfully fitted the exponential decay function under both workflows.
3. The original workflow overestimated the kinetic constant for 23 of the 47 remaining cells (49%).
4. The new workflow overestimated the kinetic constant for 21 of the 47 remaining cells (45%).
5. 3 out of the 47 (6%) remaining cells had the same kinetic constant for both workflows.

When interpreting such results, an important conclusion can be made; for a given cell population, the original workflow does not overestimate the kinetic constant for all cells. In fact, the new workflow, for 45% of the cells, outputted a kinetic constant that was on average 27% higher than the one suggested by the original workflow. However, for 49% of the cells, the original workflow outputted a kinetic constant that was on average 50% higher than the one suggested by the new workflow. It is clear that the degree of overestimation for the original workflow is much greater than the one observed with the new workflow; such element by itself would be sufficient to explain the shift of the CRRC histogram, obtained with the original workflow, to the right. At this point, we compared the kinetic curves of all the 47 remaining cells and noticed that:

1. The kinetic curves obtained with the original workflow of 9 of the 47 cells show an abnormal pattern *i.e.,* the kinetic curves clearly differ from a single exponential decay. The kinetic patterns observed in such cells (see cell IDs 6, 8, 11, 16, 25, 42, 54, 57, 60 on file '67 cells comparison.ppt') are similar to the one observed in Figure 5A, where we observe a discrepancy between the cell-contour mask and the true cell position in the original workflow. In fact, we manually tracked these 9 cells during the course of the time-lapse experiment, and they were all confirmed to be highly-motile.
2. None of the kinetic curves obtained with the new workflow show an abnormal pattern.

The above results confirmed what we observed in Figure 5 of the main text: the original workflow tends to overestimate the rate constant of substrate efflux for high-motility cells, leading to the shift of the CRRC histogram to the right (when compared to the histogram obtained with the new workflow).

Note S8: Considerations on Statistics

In our case, we were interested to compare the k_{efflux} distributions obtained from the original and new workflow, respectively. To help us choose the most appropriate statistical test, and to guide us for future experiments, we formulated a series of questions:

1) Is the data qualitative (*e.g.*, percentages, frequencies) or quantitative (*e.g.*, mean, median)
2) How many samples are we testing? If you are testing more than one group, are the groups independent or paired?
3) What is the purpose of the test? Here, there are three options: *i)* testing against a hypothesized value, *ii)* compare two (or more) populations, *iii)* establish a correlation.
4) Does the data follow a normal distribution? If yes, then use a parametric test. If not, use a non-parametric test.

We established that:

1) Our data was quantitative (k_{efflux} values)
2) Two independent groups (k_{efflux} values from the original workflow *vs* k_{efflux} values from the new workflow). Usually, two groups are considered dependent if the measurements in one group affect or are somewhat related to the measurements in the other group (*e.g.*, measuring heart pressure from the same individual before and after the administration of hypertension medications). On the other hand, two groups are considered independent if their values do not depend on each other (*e.g.*, measuring heart pressure from a group that was treated with hypertension medications and from another group that was treated with a placebo drug). In our case, although the measurements were done on the same set of cells, the groups were deemed independent because each workflow computed its own set of measurements independently.
3) The purpose is to test the two distinct k_{efflux} distributions.
4) In our case, a visual representation (Figure 6 main text) was enough to establish that our data was not normal. Sometimes, this is not possible, and an appropriate normality test is required, *e.g.*, the Shapiro-Wilks normality test.

Based on our answers, we established that the two-sample Kolmogorov-Smirnov test was appropriate to conduct our analysis. Essentially, this test estimates the probability that two groups were drawn from the same hypothetical distribution by detecting differences in both the locations (median values) and shapes (skewness values) of the distributions. The test does so by comparing the two empirical distribution functions (EDF). In general, an EDF assigns a specific probability value to every observed event in the group. The largest absolute difference between the two EDFs is referred to as the test statistic for the Kolmogorov-Smirnov test (D):

$$D = |\text{EDF}_{\text{group 1}} - \text{EDF}_{\text{group 2}}| \qquad (1)$$

where, group 1 and group 2 refer to the two independent groups being compared.

If the D value is greater than the approximate critical value (D_α) for a given α, then the null hypothesis is rejected. The null hypothesis states that the two distributions are drawn from the same probability distribution, while D_α can be calculated using the following formula:

$$D_\alpha = c_\alpha \sqrt{\frac{n_1 + n_2}{n_1 n_2}} \qquad (2)$$

where c_α is a coefficient that depends on α, and n_1 and n_2 are the sample sizes for group 1 and group 2, respectively.

Hence, if we consider our situation, the null hypothesis will state that the k_{efflux} distributions, which were obtained from two different workflows, were drawn from the same hypothetical distribution. If this were true, it would mean that the original and the new workflow process our CRRC data in a nearly identical fashion. We executed the Kolmogorov-Smirnov test using the software OriginPro. In our case, OriginPro computed $D = 0.376$ for $\alpha = 0.001$. $D_\alpha = 0.209$, and it was manually calculated from eq 2 for $c_\alpha = 1.95$, $n_1 = 365$ and $n_2 = 114$. Since $D > D_\alpha$, we concluded that the k_{efflux} distributions were not sampled from the same distribution at the 0.001 significance level. This was also confirmed by a p value of 2.82×10^{-11} ($p << 0.001$). It is noteworthy that OriginPro computes p values based on Kim and Jenrich (Kim, P. J.; Jenrich, R. I. Tables of exact sampling distribution of the two sample Kolmogorov–Smirnov criterion D_{mn} $(m < n)$ Selected Tables in Mathematical Statistics. *American Mathematical Society* **1973,** *1*, 80–129).
