# Supplementary material for: Automated identification and tracking of cells in Cytometry of Reaction Rate Constant (CRRC)
Source: PLoS One. 2023 Jul 3;18(7):e0282990. doi: 10.1371/journal.pone.0282990 (PMC10317225; doi:10.1371/journal.pone.0282990)
Supplement: Attachment — Submitted filename: Responses_to_Reviewers.pdf [file pone.0282990.s002.pdf]

We thank the Reviewers for reading the manuscript and providing constructive comments. Below our responses to the Reviewers' comments.

REVIEWER #1
Comment 1: The new CRPC workflow needs to be discussed extensively with citing relevant literature which is mainly lacking. No references have been cited in second part of introduction section line 17-36.
Response and changes made: We have added a section to the introduction to place our new workflow in the context of current literature. References 14-23 have been added to the second part of the introduction.

Comment 2: Title should be revised and can be more scientific than technical.
Response and changes made: We replace the original title with the following: "Automated identification and tracking of cells in cytometry of reaction rate constant (CRRC)"

Comment 3: All figure captions should be listed at the end and should not be scattered in the manuscript. English should be revised."
Response and changes made: Sorry. We understand that this formatting is inconvenient for reviewers; however, this is exactly what PLOS ONE requires: "Each figure caption should appear directly after the paragraph in which they are first cited".

Comment 4: English should be revised.
Response: The manuscript has been carefully proofread for English. Changes made are highlighted in the "marked-in" version of the revised manuscript.
